# Pre-treatment of composite industrial wastewater by Fenton and electro-Fenton oxidation processes

**DOI:** 10.1038/s41598-024-78846-w

**Published:** 2024-11-13

**Authors:** Basma M. Omar, Mohamed A. Zyadah, Menna Y. Ali, Mervat A. El-Sonbati

**Affiliations:** Faculty of Science, Environmental Sciences Department, Damietta University, Damietta, 34517 Egypt

**Keywords:** Multi-sourced industrial wastewater, Fenton process, Electro-Fenton process, Chemical oxygen demand, Biodegradability, Environmental monitoring, Pollution remediation

## Abstract

The present study aims to characterize three industrial wastewater samples collected from petrochemical, food and beet sugar industries to determine the pollution potential and select the appropriate pre-treatment approach. According to the biodegradability profile of the multi-sourced mixed (composite) sample, the advanced oxidation process (AOPs) namely, Fenton (F) and Electro-Fenton (EF) were adopted as pre-treatment techniques and the operating parameters such as time, type of electrodes, pH, voltage, iron and H_2_O_2_ concentrations were critically examined. Analysis of Variance (ANOVA) was conducted to compare the performance efficiency of F& EF AOPs for treating the composite samples and the total operating costs for both approaches were assessed. The results revealed that, the initial values of the composite sample were 7.11, 19.2, 32.6, 19.3, 937, 1512, 860, 3.9, 2110 and 2.34 for pH, Total Dissolved Solids (TDS), Electrical Conductivity (EC), Salinity, BOD, COD, Oil and grease (O&G), Total Phosphorous (TP), Total Suspended Solids (TSS) and Total Kjeldahl Nitrogen (TKN), respectively. In addition, EF process achieved more removal efficiency for COD, O&G, BOD, TSS, and TKN (84.3%, 69%, 85%, 72% and 71.27%) compared to Fenton which displayed 78.43%, 66%, 69%, 70.1%, and 61%, respectively. Moreover, there are statistically significant differences (*p* < 0.05) between the initial and final (pretreated) values of the composite industrial wastewater for the addressed parameters and EF was significantly (*p* < 0.05) more effective than F process. The total operating costs were 3.117 and 2.063$ for F and EF, respectively, which confirmed that EF is more efficient and cost effective than F process. It was concluded that electro-Fenton process is favorable, eco-friendly and cost-effective option for pretreating real complicated multi-sourced industrial wastewater. The present study demonstrated a new avenue for achieving efficient management of industrial wastewater generated from similar industries.

## Introduction

Water pollution is one of the most conclusive issues facing the globe, whereas the over-usage of water in various sectors such as industry, agriculture, homes, transportation, industrialization, combining with climatic changes results scarcity in water resource^1–5^. Enormous amount of wastewater containing high concentrations of organic, inorganic compounds, suspended or dissolved solids, and heavy metals were generated from various industries such as the textile, sugar, mining, battery manufacturing, iron and steel, soap and detergent, paper, chemicals, food, beverage, and petrochemical^6,7^.

The disposal of wastewater into water bodies without treatment affects human health and aquatic environment, since most of contaminants are very toxic, mutagenic, endocrine-disrupting, or potentially carcinogenic^8,9^. Globally, about 50% of child deaths and 80% of diseases are related to poor water quality^10^. Tremendous efforts have been exerted by the scientific community for minimizing the pollutant level to meet the regulatory standards for water quality and fulfill the goals of sustainable development^11^. In general, treatment approaches are adopted depending on the nature and load of pollutants^12^.

Industrial wastewater treatment techniques have been classified into physicochemical, physical, chemical, and biological processes^13^. Actually, the toxicity and recalcitrant nature of certain components in industrial wastewater, long time, and large surface area render the extensive application of conventional biological processes^9,14^. Physical processes are sometimes insufficient to achieve the discharge limits and also expensive. Moreover, physical treatments such as gravity method can only eject floating and dispersed contaminants and the membrane filtration method has a fouling drawback that limits its usage. In addition, membrane filtration and reverse osmosis are not feasible from economic point of view because they need expensive materials and the integrated methods require large time. Furthermore, secondary pollutants generation expose the environment to severe problems^6,15–17^. Chemical processes such as coagulation produces large amount of sludge due to increase some dissolved constituent’s concentration. Besides, chemical-oxidation needs transportation and storage of hazardous chemicals and has low capacity for handling wastewater^9^.

Therefore, industrial wastewater should be treated using more effective technologies which can overcome the drawbacks of the existing treatment approaches. In this context, one of the most promising techniques is the advanced oxidation process (AOP) which has great significance in environmental remediation such as organic pollutant destruction through toxicity reduction, biodegradability improvement, odor & color removal and has the advantages of being safe, controllable and efficient^6,10^. AOPs are processes that include in situ generation of highly reactive radicals for the oxidative destruction of pollutants, and can be categorized into ozonation, UV ozonation, ultrasound, wet air oxidation, photocatalysis, persulfate oxidation, Fenton and Fenton-like processes^4,6,18,19^.

The Fenton and electro-Fenton techniques superior other processes as they can be controlled, has high efficiency for contaminant destruction, H_2_O_2_ generation, ability to operate in mild conditions^3,20^. Compared with other AOPs, the Fenton process is the most popular due to high performance, non-toxicity, and simplicity. This process based on the catalytic decomposition of H_2_O_2_ into •OH by Fe, and non-selectively enable the destruction of organic compounds^21^. Moreover, both ferrous and ferric ions generated are coagulants and the formed hydroxide compound can act as adsorbent. Consequently, it has three functions in the treatment process; oxidation, coagulation, and adsorption^22^. Electro-Fenton processes have attracted high attention in industrial wastewater treatment due to the efficient capability of fractionating a number of organic compounds. In this process, a continuous electro-generation of H_2_O_2_ predominates at a proper cathode with the addition of an iron catalyst to generate high oxidant hydroxyl free radical (•OH) in the bulk of the solution^23^. Previous researches revealed the efficacy of electro-Fenton process using platinum coated titanium (Pt/Ti) electrodes, aluminum electrodes, and Ti/Pt anode and graphite cathode for composite industrial wastewater treatment^24,25^.

The complexity of composite (mixed multi-sourced) industrial wastewater generated from various small-scale industries and high uncertainty in both quantity and quality represent challenges for the traditional methods to successfully treat such complicated wastewater^25^. The main aim of the present study is to resolve problem of drainage the multi-sourced industrial wastewater without proper treatment which has negative impact on the community of Damietta and Dakahlia cities, Egypt, and this was achieved via collection and characterization of representative composite sample from food, beet sugar, and petrochemical industries, investigate the biodegradability, and the feasibility of Fenton and electro-Fenton oxidation processes for effective pretreatment. The effect of various operational parameters on both Fenton and electro-Fenton oxidation techniques were considered.

## Materials and methods

### Sampling

Industrial wastewaters samples were collected by grab method from a petrochemical, beet sugar and food plants located in industrial zones of New Damietta and Dakahlia city- Egypt, in pre-washed polyethylene containers, cooled to 4 °C using ice boxes and acidified by HNO_3_ or HCl to ~ pH 2.0 until were received by the laboratory to avoid any changes in the chemical composition of samples^26^. Composite samples were obtained by mixing equal ratios of the previously collected samples. All the chemicals and reagents used during the experiments were of analytical grade without further purification.

## Materials

Pure analytical grade materials, such as ferrous sulfate heptahydrate (FeSO_4_.7H_2_O, Alpha Chemika, FS186) and H_2_O_2_ solution (Almasria for Chemicals, 30% w/w) were utilized in all experiments. In addition, pH was adjusted using 1 N of both sodium hydroxide and sulfuric acid (Almasria for Chemicals).

## Experimental setup

**Fig. 1:**
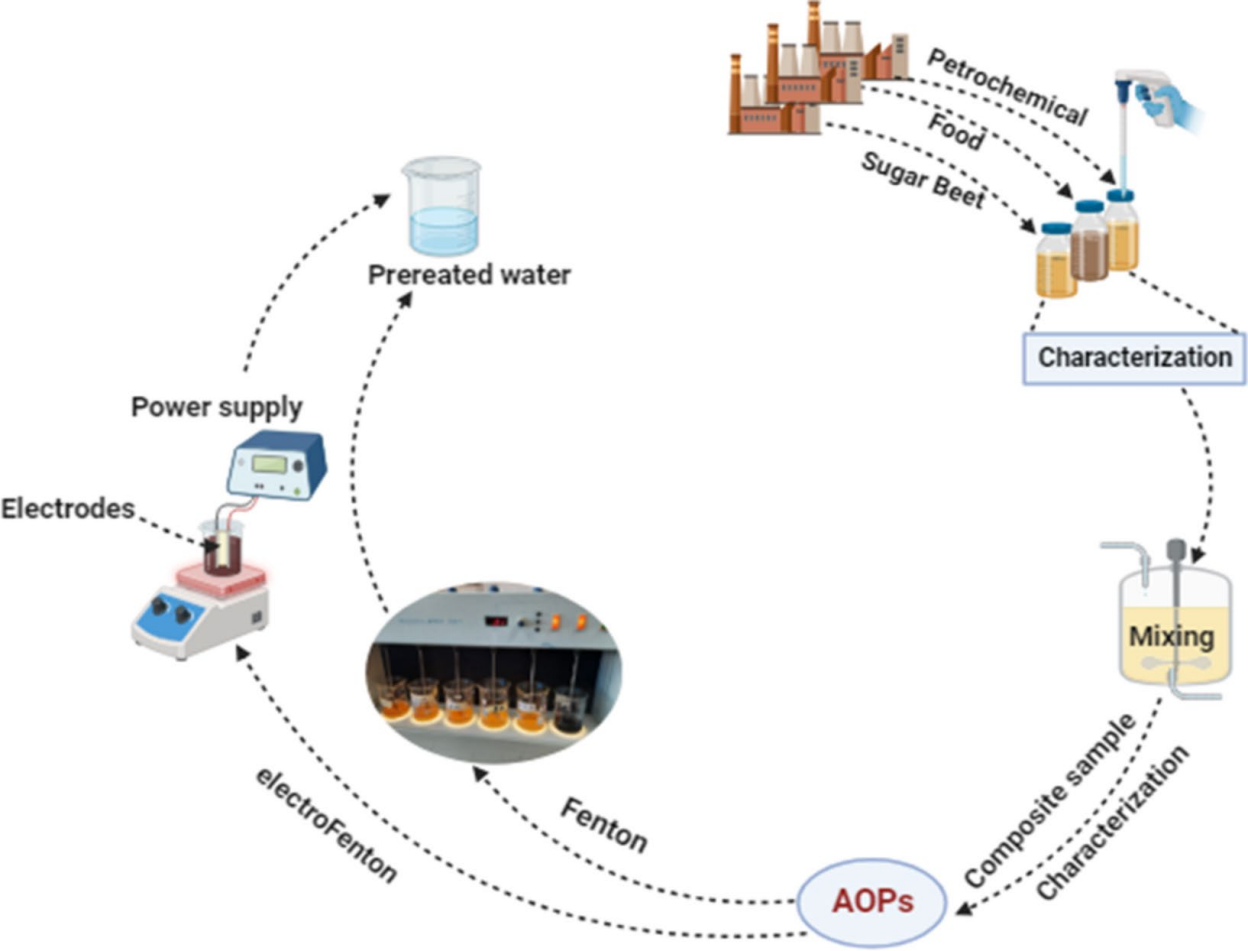
Schematic diagram for set up of the whole processes.

All the wastewater samples were characterized for some physicochemical parameters such as pH, EC, salinity, TSS, TDS, DO, BOD, COD, O&G, TKN, and TP according to standard method^26^. The present study assessed the biodegradability of industrial wastewaters during 28 days (BOD determination). Subsequently, the sample was treated by Fenton and electro Fenton processes at different operating parameters, where the COD parameter was used as an indicator for the performance efficiency of the treatment processes (Fig. 1).

## Fenton process

In Fenton process, the hydroxyl radicals (^•^OH) oxidize organic matter in industrial wastewater by free radical reaction and electron transfer^27,28^. 100 ml of the composite industrial wastewater sample was treated at different operating parameters such as pH (3–11), Fe^2+^ (0.5, 1, 2, 4, 8 mg/L), H_2_O_2_ (0.05, 0.1, 0.2, 0.3, 0.5 g/L). All experiments were performed in a jar test apparatus^29,30^.

## Electro-Fenton (EF) process

Electro-Fenton (EF) process is a considerable AOPs in industrial wastewater treatment due to ability to generate hydrogen peroxide, lower catalyst requirements, high mineralization competence, and regeneration of Fe^2+^ at the cathode according to the following Eqs. (1,2) that oxidize and destruct the contaminants in the industrial wastewater^20,25^.

1 $$                      O_{2}+2H^{+}+ 2e^{--}\rightarrow H_{2}O_{2}$$

2 $$\:{Fe}^{2+\:}+H_{2}O_{2}\:\to\:{Fe}^{3+}\:+OH^{-}+\: \cdot OH\:$$

1000 ml of the investigated composite industrial wastewater samples was put into 2 L capacity beaker, two electrodes with dimensions of 5*8 cm (length x width) were immersed with an effective surface area of 9 cm^2^. Different parameters such as pH (3–11), electrodes (iron/ iron, stainless-steel/ stainless-steel, and iron/stainless-steel) at different time intervals (0, 20, 30, 60, 90, 120 min), iron (0.5, 1, 2, 4, 8 mg/L), hydrogen peroxide (0.05, 0.1, 0.2, 0.3, 0.5 g/L), voltage (0.5, 1, 2, 3, 4 V) were investigated and COD parameter was adopted as an efficiency indicator for the treatment process.

### Methods and instruments

The pH of the samples was measured by pH/Mv/ISE/temperature bench Meter (model, AD1020Adwa). Salinity, EC and TDS were examined by digital meter (Digital Portable TDS/ Conductivity meter Model, 4520 JENWAY). TSS was determined by filtration and evaporation at 103–105 °C oven (lab index). Determination of DO and BOD were carried out using (Lovibond BOD system model, BD600) by the Respirometric Method at 20 °C. TP was determined by the Persulfate method using autoclave (model, DSX-280) and spectrophotometer (model, 7305 JENWAY). Oil and grease were determined by Liquid-Liquid, Partition-Gravimetric method. Phenate method is used to quantify the TKN by (DKL fully automatic digestion units and UDK139 semi-automatic distillation unit (Model, VELP SCIENTIFICA)^26^. The COD was measured using quick COD lab (model, 03E4318 LAR) under recommended conditions (temperature 900 °C)^31^.

The biodegradability of the three collected industrial wastewater and composite samples was assessed during 28 days through BOD determination (Lovibond BOD system model, BD600), where a very small amount of a seed material and nutrients was added to the solution. A jar test apparatus (flocculator sw1) was used for Fenton treatment process while, electro-Fenton process performed by power supply model (photon DC power supply (0-15v) /2A and cjj79-1 magnetism heating mixer) in the presence of suitable electrodes. All measurements were carried out in triplicate and the average values were considered in the calculation^11^.

The %COD removal was expressed by the following equation:

3 $$\:\%COD\:removal=\frac{(\text{C}\text{O}\text{D}\text{i}-\text{C}\text{O}\text{D}\text{f})}{\text{C}\text{O}\text{D}\text{i}}\times\:100$$

Where (COD_i_) and (COD_f_) are initial and final COD values, respectively.

Analysis of Variance (ANOVA) was performed to compare the performance efficiency of F& EF AOPs for the pretreated industrial wastewater using SPSS package (version 25), the regression analysis was performed by program Statistica 8.

## Cost analysis

The total operating cost (OC) was computed through the sumption of the prices of electrical energy ($kWh^− 1^), electrode material ($kg^− 1^), and chemicals, along with other expenses for labor, maintenance, etc. The Eq. (4) was utilized to obtain the OC based on the elimination of 1 kg of COD per m^3^ of treated composite effluent^23^.

4 $$\:OC=\text{a}\text{C}\text{E}\text{N}+\text{b}\text{C}\text{E}\text{L}+\text{cCCH}+\text{other}\:\text{costs}$$

Where OC is the operational cost ($ m^− 3^ kg^− 1^ COD removal), CEN (kWh.m^− 3^ kg^− 1^ COD removal), CEL (kg.m^− 3^ kg^− 1^ COD removal) and CCH (kg.m^− 3^ kg^− 1^ COD removal) are the consumption of electrical energy, electrodes and chemicals, respectively. While, a, b and c are the prices of electrical energy ($kWh^− 1^), electrode material ($ kg^− 1^) and chemicals ($ kg^− 1^), respectivel**y.** Equation (5) was used to calculate CEN

5 $$\:CEN=\frac{\text{U}\text{*}\text{I}\text{*}\text{t}}{{C}_{MO}\text{*}\text{X}\text{*}\text{V}\text{*}1000}$$

Where U (V), I (A), t (h), C_MO_ (kgm^− 3^), X and V (m^3^) are the applied voltage, current flow, reaction time, initial COD value, fraction removal for the treatment time and volume of composite sample, respectively. CEL was calculated by Eq. (6) using Faraday’s law:

6 $$\:CEL=\frac{\text{M}\text{w}\text{*}\text{I}\text{*}\text{t}}{{C}_{MO}\text{*}\text{X}\text{*}\text{z}\text{*}\text{F}\text{*}\text{V}}$$

Where M_w_ is the electrode molecular mass (kg mol^− 1^), z is the number of electrons transferred (ZFe = 3), F is the Faraday’s constant (96487 C mol^− 1^) and t is the reaction time (s)^23^.

## Results and discussion

### Characterization of industrial wastewater samples

Table 1 displayed the characterization of the industrial wastewater samples compared with Egyptian standards (ES); Law 92/2013^32^ for drainage into marine environment and Nile branches, respectively. The results revealed that the pH ranged from 4.84 to 7.51, while TDS, EC and salinity ranged from 0.77 to 40.4 g/L, 1.29 to 78.4 ms/cm and 0.8 to 60.5 g/L, respectively. Moreover, oil and grease were in the range of 139 to 860 mg/L, and TSS ranged from 404 to 2733 mg/L. In addition, TP, TN and COD varied from 0.15–4.24 mg/L, 1.21–4.93 mg/L and 1320.3 to 1526.9 mg/L, respectively. It is clear from table 1 that the values of the investigated parameters exceeded the Egyptian standards (ES); Law 92/2013^32^ for drainage into marine environment and Nile branches. Consequently, it is a must to treat the industrial wastewater to comply with the Egyptian laws and protect the aquatic environment from deterioration.

The results obtained in the present study were nearly matched with that recorded by Shamsan et al.^2^ who characterized food processing industry wastewater and found that pH value was 6.68, conductivity 3737 *µ*s/cm, oil and grease 52.8 mg/L, BOD 1010 mg/L, COD 2820 mg/L, TSS 1050 mg/L, total nitrogen 154 mg/L, and total phosphorous 18 mg/L. Nidheesh et al.^25^ analyzed mixed industrial wastewater and reported that the values of pH, TSS, conductivity, BOD and COD were 7.8, 0.26 g/L, 13.31ms/cm, 239 mg/L., and 795 mg/L, respectively. In addition Xu et al.^33^ examined sugar processing industry wastewater and showed that pH, COD, TSS, conductivity values ranged between 6.8–7.2, 20–22 g/L, 480–520 mg/L, and 28–30 ms/cm, respectively. The characterization of sugar processing industry wastewater for COD, total phosphorous, conductivity, and TDS were 3960 mg/L, 17.13 mg/L, 3580 *µ*s/cm, and 1760 mg/L, respectively^7^. Moreover, Ahmed et al.^34^ investigated petrochemicals industry wastewater and found that pH was 7.4 ± 0.5, TSS 53.4 ± 48.5 mg/L, COD 532.9 ± 528.6 mg/L, BOD 315.8 ± 307.6 mg/L, TN 43.5 ± 21.1 mg/L, and TP 7.0 ± 4.1 mg/L.

**Table 1:** The characterization of the industrial wastewater samples compared with Egyptian Standards (ES); Law 92/2013 for drainage into marine environment and Nile branches^32^, respectively.

Parameter	Unit	Food	Petrochemical	Sugar	Composite	Law92/2013			
						The Nile River to the Delta (mg/L) except pH	Surface water mg/L) except pH	Damietta-Rashid Branch mg/L) except pH	Agriculture use mg/L) except pH
pH	–	4.84	6.96	7.51	7.11	6–9	6.5–8.5	6–9	6.5–8.5
TDS	g/L	0.78	40.4	2.39	19.2	1200	≤ 500	800	≤ 1000
EC	ms/cm	1.29	78.4	4.05	32.6	–	–	–	–
Salinity	g/L	0.8	60.5	1.8	19.3	–	–	–	–
BOD	mg/L	720.2	453.3	1070	937	30	≤ 6	20	≤ 30
COD	mg/L	1320.3	684.6	1526.9	1512	40	≤ 10	30	≤ 50
BOD/COD	–	0.55	0.66	0.70	0.62	–	–	–	≤ 3
O & G	mg/L	139	242	2235	860	5	≤ 0.1	5	3
TP	mg/L	1.54	0.15	4.24	3.9	1	≤ 2	1	–
TSS	mg/L	404	2733	1413	2110	30	–	30	15
TKN	mg/L	1.21	2.73	4.93	2.34	5	≤ 3.5	5	6.5–8.5

### The biodegradability of industrial wastewaters samples

The present study assessed the biodegradability of the composite and the three individual industrial wastewaters (petrochemical, food and beet sugar) samples collected from industrial zone of New Damietta and Dakahlia City, Egypt within 28 days and were categorized according to the classification displayed in Table 2^35^.

**Table 2:** Biodegradability categorization.

Days	Biodegradability classification
Within 5 days	Easily biologically degradable
Between 5 and 21 days	Medium biologically degradable
Between 21 and 25 days	Moderately biologically degradable
Within 28 days	Maximum inert

**Fig. 2:**
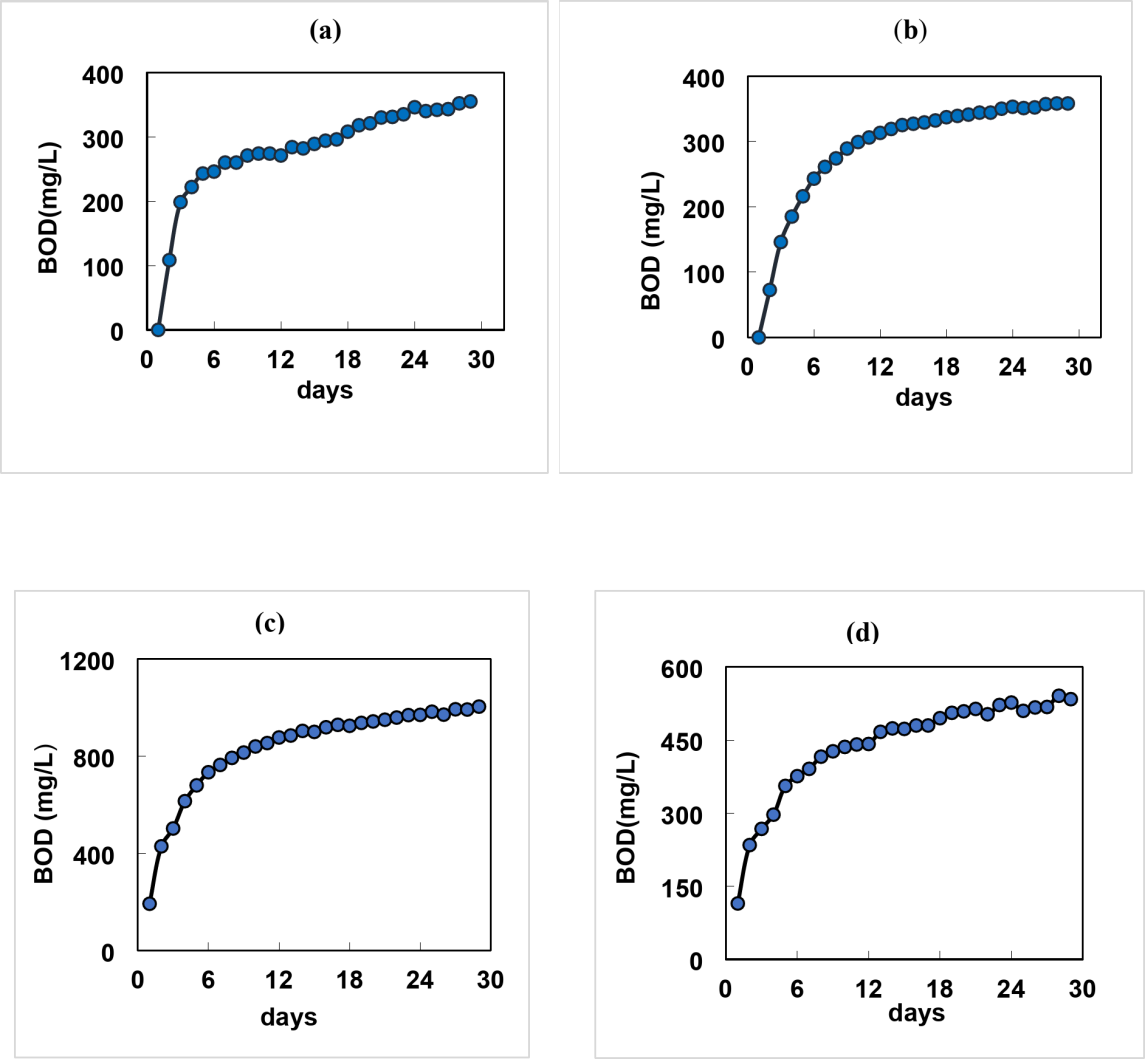
The biodegradation curves of industrial wastewaters samples (**a**) petrochemical, (**b**) food, (**c**) beet sugar, and (**d**) composite samples within 28 days.

The biodegradation curves obtained for the samples under investigation are shown in Fig. 2. It is clear that the plateau area of petrochemical industry sample was reached after about 23 days (Fig. 2a) which implied moderately biologically degradable industrial wastewater. While plateau region for food, beet sugar and mixed samples were about 19, 16 and 19 days, respectively (Fig. 2b, c, d) indicating medium biodegradability.

### Treatment of composite industrial wastewater sample

As the biodegradability results of the industrial wastewater samples ranged from medium to moderate, therefore the chemical treatment was adopted rather than biological technique and the composite sample was selected for subsequent treatment.

### Advanced oxidation process (AOP) by Fenton’s reaction

**Fig. 3:**
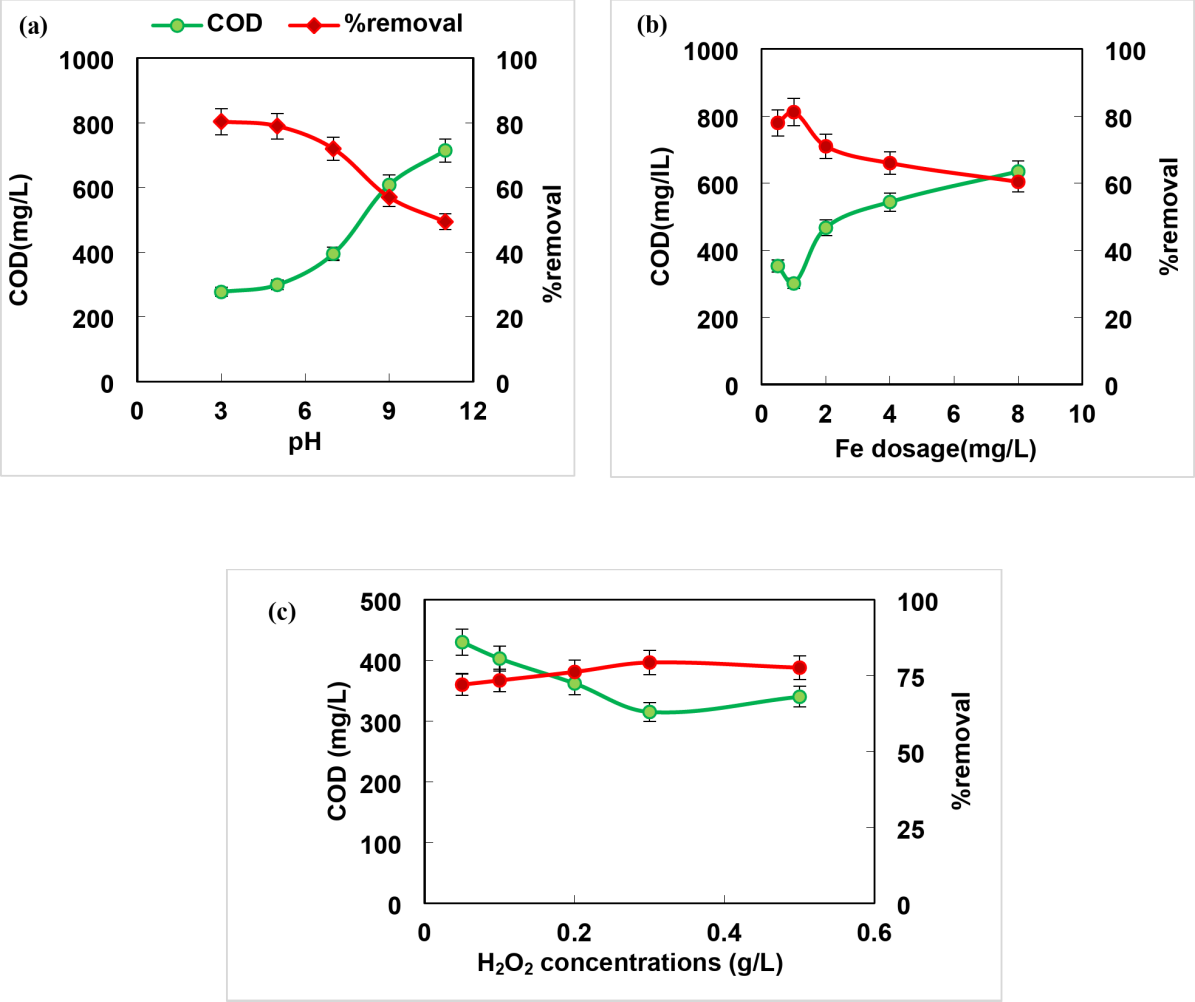
Effect of different operating parameters on COD removal of mixed samples by Fenton process (**a**) pH, (**b**) dose of iron and (**c**) dose of hydrogen peroxide (pH 3, ferrous ion concentration 1 mg/L, and hydrogen peroxide concentration 0.3 g/L).

The Fenton process is a promising eco-friendly treatment technique applied to reduce and eject persistent organic pollutants from wastewater^1^. The pH is the key factor that greatly influences the efficiency of the process. The hydroxyl radicals were generated by reaction of H_2_O_2_ with iron without any iron precipitation. As shown in Fig. 3a, the COD removal efficiency was 80.4%, 79%, 72%, 57% and 49.4% for the pHs 3, 5, 7, 9, 11 respectively. It is clear that increasing pH value than 4 led to decrease the efficiency of Fenton process due to the production of iron oxyhydroxide and the deposition of iron as ferric hydroxide at pH 4 or ferrous hydroxide at pH 7. At lower pH (≤ 2) the instability of hydrogen peroxide was reported^36^, therefore, pH 3 was selected as an optimum for sample treatment in the subsequent work. The obtained results were similar to that recorded (89% COD) by Younes & Al-Sa`ed^37^ at the optimum conditions (pH 3, H_2_O_2_/Fe^+ 2^ 10:1, and COD 15400–18200 mg/L) for pretreatment of mixed wastewaters. Cheng et al.^38^ pretreated petrochemical wastewater at pH 3 and achieved 89.8% COD removal, BOD_5_/COD increased from 0.052 to 0.62 after 60 min, 120 mg/L Fe^2+^ and 500 mg/L H_2_O_2_.

The iron (Fe^2+^) concentration plays an effective role in the Fenton process ​​as a catalyst because it influences the performance efficiency, reaction time, and sludge generation. It is showed that the COD removal percentage increased from 60.4 to 81.2% with increasing ferrous ion dose up to 1 mg/L then decreased (Fig. 3b). This can be interpreted as the over dosage of Fe^2+^ not only increases economic costs and iron sludge generation, but also promotes the scavenging effect of OH radical^8^. Therefore, the optimum concentration of ferrous ion that enhances maximum removal of COD was adopted as 1 mg/L which more less than that applied (60 mg/L Fe^+ 2^) by Delil and Gören^39^ with maximum COD removal of 76% (initial COD 6200 mg/L) at pH 2, and 250 mg/L H_2_O_2_ in treatment of sugar industry wastewater; and 120 mg/L Fe^+ 2^ by Cheng et al.^38^ who used Fenton process to pretreat petrochemical wastewater and achieved 89.8% COD removal after 60 min and 500 mg/L H_2_O_2_.

H_2_O_2_ plays a pivotal role in the Fenton process, as it is responsible for the generation of the hydroxyl radical^22^. The effect of hydrogen peroxide concentration (0.05–0.5 g/L) on COD removal percentages was displayed (Fig. 3c). It was observed that increasing H_2_O_2_ concentration stimulates pollutant decomposition, however H_2_O_2_ overdose sweeps and converges the generated hydroxyl radicals^22,36^. Thus, 0.3 g/L H_2_O_2_ was adopted as an optimum dose in the sequent work. The obtained results are consistent with those achieved by Delil and Gören^39^and Cheng et al.^38^ who recorded a maximum COD removal of 76% and 89.8% using 250 and 300 mg/L H_2_O_2_ for treatment of sugar and petrochemical wastewater, respectively. Suhan et al. 2021^22^ investigated the Fenton degradation of Sudan black B (SBB) dye in an aqueous solution at the optimum conditions (150 mg/L Fe^2+^, 100 mg/L H_2_O_2_, and 30 min) and achieved *>* 80% COD removal.

### Advanced oxidation process (AOP) by Electro-Fenton’s reaction

**Fig. 4:**
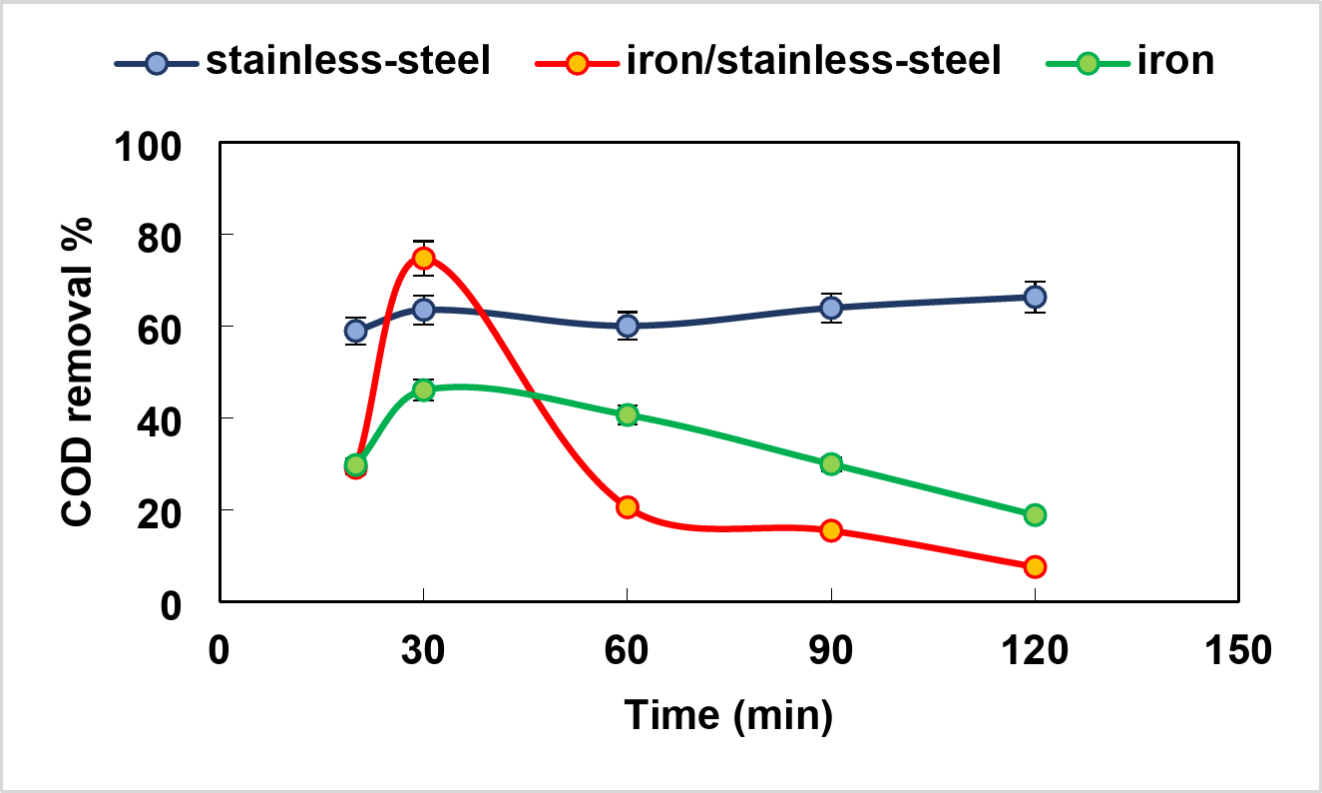
Effect of electrodes type on COD removal % of composite industrial wastewater by Electro-Fenton process at different contact time (pH 5, H_2_O_2_ concentration 0.3 g/L, 1 mg/L ferrous ion dose).

The type of electrodes is a significant parameter that affects the electro-Fenton process^40^. The influence of electrode’s type on the removal efficiency of COD of composite industrial wastewater samples were critically investigated at time intervals (0, 20, 30, 60, 90,120 min). The results presented in Fig. 4. showed that the COD removal percentage using stainless steel / stainless steel electrodes increased to 63% by increasing time to 30 min then decreased steadily. While, employing iron/ iron and stainless steel/ iron electrodes, the COD removal percentage increased to 46.1% and 74.8%, respectively by increasing time to 30 min then sharply decreased and this may be attributed to the ease of iron oxidation and the higher energy required for the treatment process^41^. Therefore, the optimum electrodes that achieved maximum removal of COD was stainless-steel/ stainless-steel that is used in the subsequent work at 30 min as an optimum time. The obtained results are higher than that reported by Nidheesh, et al.^25^who treated mixed industrial wastewater (BOD/COD 0.3, initial COD 795 mg/L) using platinum coated titanium electrodes at pH 3, 3 V and 60 min with maximum % removal of 54.57. In addition, Popat et al.^24^treated mixed industrial wastewater with initial COD value 1152 mg/ L achieving COD removal percent of 60% at 10 V, 1 cm electrode distance, 25 cm^2^ surface area, 10 mg /L catalyst dosage and 60 min contact time using Ti/Pt anode and graphite cathode.

**Fig. 5:**
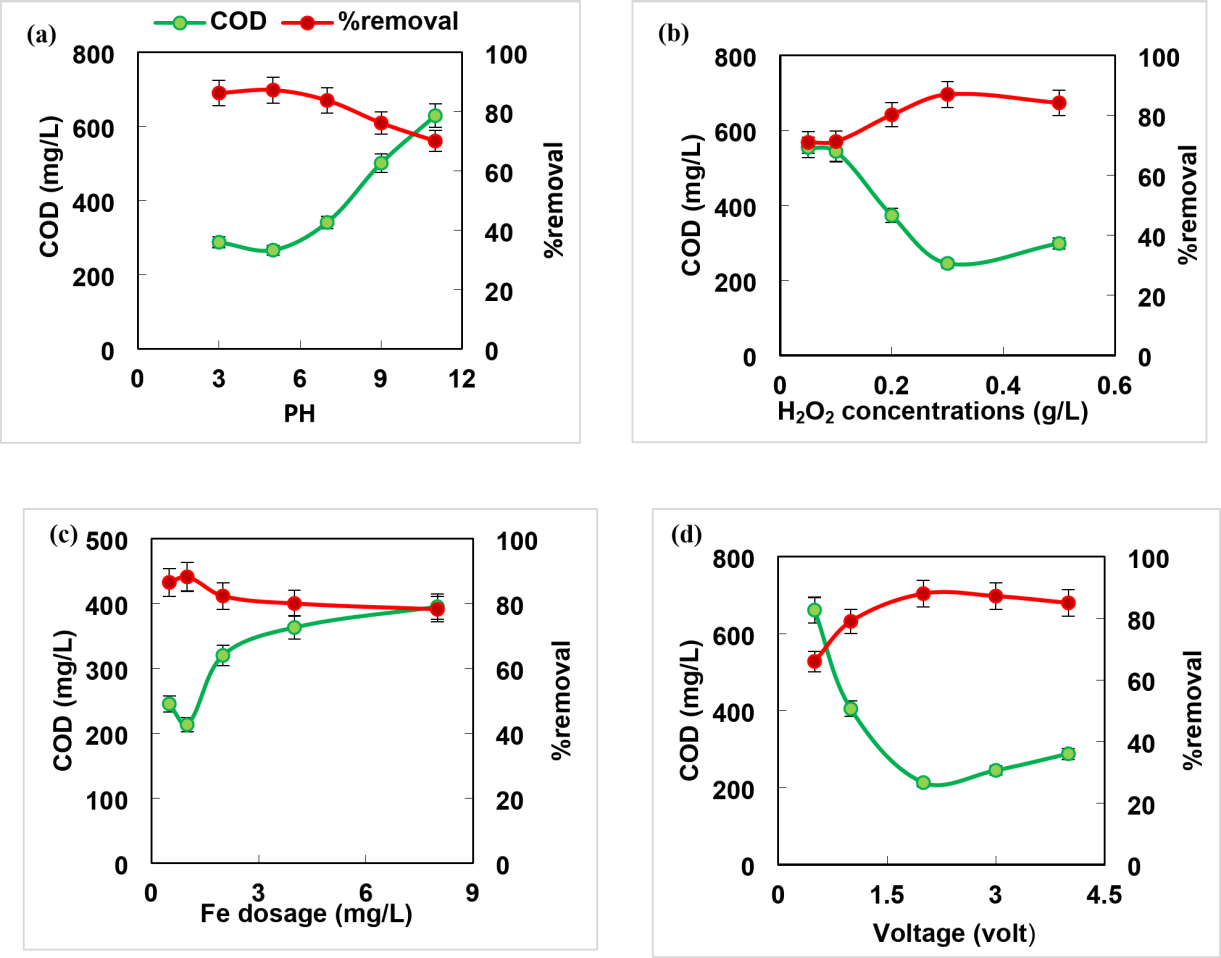
Effect of different operating parameters on COD removal of composite sample by Electro-Fenton’s process (**a**) pH, (**b**) H_2_O_2_ concentration, (**c**) iron dose, (**d**) voltage (employing stainless-steel electrode).

pH is a significant factor which affects the performance efficiency of electrochemical reactions during wastewater treatment. It’s clear from Fig. 5a that 86.3–87.3% COD removal percentage was obtained at pH 3–5 as in acidic conditions which favors H_2_O_2_ generation due to the reduction of dissolved oxygen to H_2_O_2_ by protons, while a very low pH catalyzes the evolution of hydrogen gas which decomposes H_2_O_2_ and decreases the number of active sites for H_2_O_2_ generation^3^, Thus, pH 5 is adopted as an optimum in the subsequent work. These results are similar to that recorded by Nidheesh et al.^25^and are not compatible with that reported by Nidheesh et al.^41^ who found that COD and color removal was 55% and 99.8%, respectively at pH 7.7 and 60 min electrolysis time at initial COD value 1727 mg/ L using graphite plates in treatment of mixed industrial wastewater.

The concentration of H_2_O_2_ is a crucial factor in electro-Fenton processes due to the ability to decompose organic pollutants. Figure 5b displayed that COD removal percentage increases from 71 to 87% with increasing the concentration of hydrogen peroxide from 0 to 0.3 g/L, and this may be attributed to the acceleration of hydroxyl radical generation^3,40^. This behavior is inconsistent with that reported by Delil and Gören^39^who revealed that COD removal efficiency declined with increasing H_2_O_2_ concentration as the free radical hydroperoxyl (^●^HO_2_) with the lower oxidation power produced can hindered the effect of hydroxyl radicals. Moreover, Suhan et al.2020^23^ addressed the treatment efficiency of synthetic textile wastewater using EF process and achieved a maximum COD removal (76.8%) at 2mM Fe^2+^ to 1000 ppm H_2_O_2_ ratio.

As clear from Fig. 5c the COD removal percentage increased to 88.2% by increasing ferrous ion dose up to 1 mg/L then decreased, and this may be due scavenging of OH radicals by higher Fe^2+^ concentration^39^. The extent of elimination may become constant by increasing Fe^2+^ concentrations due to Fe^2+^ consumption of ∙OH^23^. Popat et al.^24^ achieved COD removal percent of 60% using catalyst dosage of 10 mg /L for treating mixed industrial wastewater. In addition, Nidheesh et al.^25^showed a significant COD reduction with addition of iron source in treatment of mixed industrial wastewater due to enhancement the production of hydroxyl radical (^•^OH).

The application of high voltage leads to raising current density which is an important parameter resulted in an increase in hydrogen peroxide production, increase in OH• and accelerate iron ions regeneration, therefore enhances the performance of treatment process^40^. As presented in Fig. 5d, the COD removal percentage increased to 88% by increasing voltage up to 2 volts then slightly decreased. This similar to that obtained by Nidheesh et al.^25^ who indicated that the applied 3 V is preferable than 5 V in treatment of mixed industrial wastewater (initial COD 795 mg/L, platinum coated titanium electrodes and 60 min) from energy consumption view point which decreases operating costs and is a favored option for large scale application. In addition, Nidheesh et al.^41^ showed that the volume of the sludge is increased with rising voltage more than 4 V in the treatment of mixed industrial wastewater by electrochemical oxidation process (initial COD value 1727 mg/ L, pH7.7,60 min using graphite plate) and found that COD and color removal was 55% and 99.8%, respectively.

### Estimation the performance efficiency of F and EF treatment processes for composite industrial wastewater treatment

**Fig. 6:**
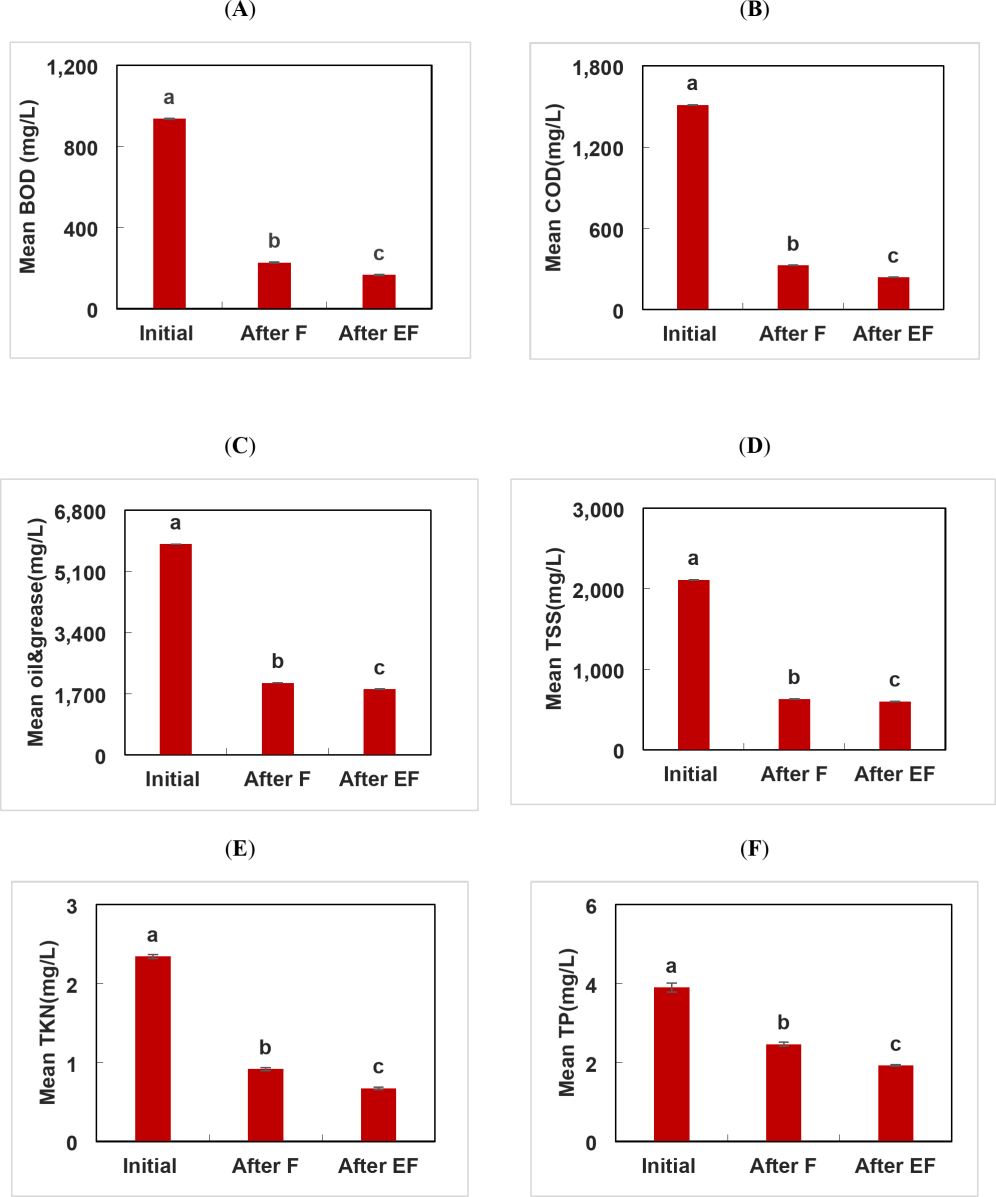
Estimation the performance efficiency of Fenton and electro-Fenton treatment processes for composite industrial wastewater treatment (**A**) BOD, (**B**) COD, (**C**) Oil and grease, (**D**) TSS, (**E**) TKN, (**F**) TP. Different letters denote significant difference (*p* < 0.05) based on one way ANOVA analysis where a > b > c.

**Fig. 7:**
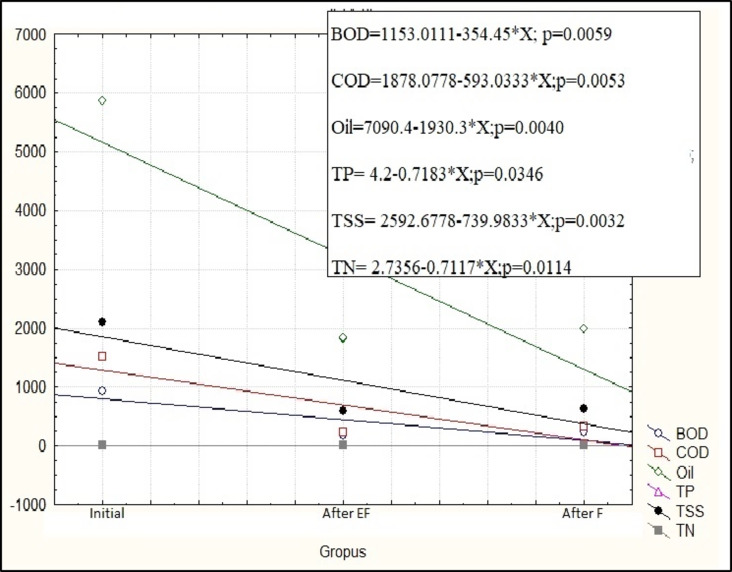
Regression analysis of multi variables (BOD, COD, Oil and grease, TP, TSS, and TN) against initial, EF and F values.

A one-way Analysis of Variance (ANOVA) is a statistical powerful technique used to analyze the differences between two or more groups^42^.The values of the addressed parameters recorded p-value less than α value of 0.05, where α indicates a 5% deviation of the mean values, are considered reliable (significant) to be used in the statistical analysis. The results of ANOVA and regression analysis are displayed in Figs. 6 and 7, it is clear that the parameters probability values indicate statistically significant differences (*p* < 0.05) between the initial and pre-treated composite wastewater samples. In addition, EF was significantly (*p* < 0.05) more effective than F process in accordance with the physicochemical analysis of the composite industrial wastewater sample (Table 3). The results illustrated that EF process achieved removal efficiency for COD, O&G, BOD, TSS, and TKN, by 84.3%, 69%, 85%, 72% and 71.27% compared to Fenton which displayed 78.43, 66, 69%, 70.1, and 61%, respectively. The obtained results matched with that recorded by Kumar et al.^43^ who used EF to treat composite sample from chemicals and textile industries and achieved 77.7% COD removal. In addition, Nidheesh et al.^25^ treated composite sample from chemical plants, oil, cotton textile, rubber and plastic and recorded ~ 55% removal of COD by EF process.

**Table 3:** Estimation the performance efficiency of Fenton and electro-Fenton treatment processes for composite industrial wastewater.

Parameter	Unit	Initial	After AOPs			
			EF	Removal%	F	Removal%
pH	–	7.11	5.3		3.2	
TDS	g/L	19.2	18.6	3.1	18.3	4.6
EC	ms/cm	32.6	–	28.7	–	28.5
Salinity	g/L	19.3	18.4	4.6	18.1	6.2
BOD	mg/L	937	167	82.2	228.2	75.6
COD	mg/L	1512	238	84.3	326	78.43
O&G	mg/L	5860	1830	69	2000	66
TP	mg/L	3.9	1.92	51	2.46	37
TSS	mg/L	2110	598	72	630	70.1
TKN	mg/L	2.34	0.67	71.27	0.92	61

### Operating cost analysis for F and EF processes at optimum conditions

Costs are one of the crucial issues to advance and bring F& EF AOPs processes to industrial scale. The main purpose of cost analysis is to evaluate the suitability of a technology to be an alternative for conventional approaches^44,45^. It is obvious from Table 4 that operating costs include power, chemical (H_2_O_2_ and Fe^2+^) & electrodes consumption and other costs (maintenance and labor). The maximum COD removal for F and EF techniques at the optimum operating conditions were 78.43% and 84.30%, with total OC of 3.117 and 2.063 $, respectively. However, the cost of power and iron of F is less than that for EF process, the latter is better from different aspects such as more removal efficiency, low sludge volume and regeneration of both H_2_O_2_ and Fe^2+^ throughout the process. Thus, EF is considered as an effective platform to treat industrial wastewaters whenever the cost criteria will be one of the major concerning factors.

**Table 4:** F and EF processes performances and cost analysis at optimum conditions.

Process	Optimum conditions	COD removal %	Cost analysis Basis: 1 kg COD removal per m^3^ composite industrial wastewater					
			aCEN, ($)	bCEL, ($)	cCCH, ($)		Other costs, ($)	OC, ($)
					Fe^+^	H_2_O_2_		
F	H_2_O_2_ and ferrous ion dose 0.3 g/L, 1 mg/L and stainless-steel electrode, (pH 3–5)	78.43%	0.0016	00.0	1.83 × 10^− 5^	3.06	0.055	3.117
EF		84.30%	0.02	1.3 × 10^− 5^	5 × 10^− 5^	1.98	0.063	2.063

### Comparison of maximum COD removal percentages with previous studies

**Table:** Table 5 displayed a comparative study of the Fenton and electro-Fenton processes with previous studies^24,25,37,41,43,46^ for the pretreatment of composite samples at optimum operating conditions (pH, voltage, electrode type, Fe^2+^ and H_2_O_2_ concentrations). The current study achieved maximum COD removal percentage 84.3% and 78.43% for electro-Fenton and Fenton process, respectively. It is clear that both processes revealed good performance efficiency for pretreatment of the addressed samples compared to other studies which are unacceptable from economic and environmental view point due to adopting expensive electrodes or combining more than one technique and generation of high amount of sludge that limit their applicability on industrial scale.

Type of wastewater	Treatment technique	COD Removal %	Operating Conditions	Reference
Composite sample (food, Petrochemical, beet sugar)	EF F	84.3% 78.43%	H_2_O_2_ and ferrous ion dose 0.3 g/L, 1 mg/L and stainless-steel electrode, (pH 3–5)	Current study
Mixed Waste Chemicals	F	78.41%	Initial COD 2676 mg/L, [H_2_O_2_]: [Fe^2+^] = 4.5:1 and pH 4	^46^
Composite sample (chemical, textile industries)	EF	77.7%	1 h of electrolysis, 1 V, pH 3 and 50 mM hydrogen peroxide	^43^
Composite sample (textile, chemical industries)	EF	55%	Initial COD 1727 mg/L, graphite electrodes, 1 h electrolysis, pH 7.7, voltage 4 V, and NaCl dose 1 g/L.	^41^
Composite sample from agro-food industrial wastewaters	F	33%	H_2_O_2_/COD (w/w2:1), H_2_O_2_/Fe^+ 2^ (w/w10:1), initial COD15400-18200 and pH 3, and settling (2 h).	^37^
Composite sample (chemical plants, oil, cotton textile, rubber, plastic)	EF	54.57%	Initial COD 795 mg/L, laterite (catalyst) dosage 10 mg/L, platinum coated titanium electrodes after 60 min of treatment, voltage 3 V, pH 3	^25^
Composite sample (pharmaceutical, dyes, textile industries)	EF	60%	initial COD 1152 mg/L, voltage10 v, electrode distance 1 cm, pH 3surface area of 25 cm^2^, catalyst dosage of 10 mg /L ,1 h and persulphate dosage of 200 mg /L using Ti/Pt anode and graphite cathode	^24^

Table 5. Comparative study of maximum COD removal percentages after Fenton and Electro- Fenton processes with previous studies.

## Conclusion

Increasing water shortage and rigorous local and regional legislation on industrial effluents obliged the industries to reduce contamination through employing advanced treatment techniques and reuse the treated water in other activities. The current study was conducted to characterize three industrial wastewater samples (petrochemical, food, beet sugar) and their composite, followed by assessment of their biodegradability to adopt the proper treatment approach. Moreover, the performance efficiency of the Fenton and electro-Fenton processes as the selected techniques were evaluated at various operating conditions (pH, H_2_O_2_ concentration, Fe^2+^ dose, type of electrode, and applied voltage). The obtained results showed that the optimum conditions for Fenton process were 3 for pH, 0.3 g/L H_2_O_2_ 1 mg/L Fe^2+^ and 30 min settling time, while for electro-Fenton were 2 V, 3–5 pH, 0.3 g/L H_2_O_2_ and 1 mg/L Fe^2+^ using stainless-steel / stainless-steel electrodes. The results revealed that the EF process using stainless steel electrodes was more efficient (*p* < 0.05) and cost effective (one and half times lower) than F process for the treatment of the addressed composite samples. However, the characterization of the treated sample by both approaches was higher than the discharge standards, thus post-treatment is required and the pH should be neutralized before discharging. In conclusion, pre-treatment methodology should be implemented according to the type and concentration of contaminants in the industrial wastewater and the treated water can be safely reused or disposed in the aquatic environment and the generated sludge may be used as fertilizer for agricultural crops.

### Future perspectives

The application of EF-based AOPs in industrial wastewater remediation is expected to develop in response to emerging challenges and advancements in technologies. Several future perspectives may be taken into consideration to pave the way for enhancement for the recent technologies.

- **Combination with other simple and cost-effective techniques**: integrating EF AOPs with adsorption technique using agro-waste based adsorbents can improve the overall environmental profile of the treatment process.
- **Employment of renewable energy sources**: adopting renewable energy sources can promote energy efficiency, minimize energy consumption and environmental footprint of industrial wastewater treatment.
- **Enhancement the performance of the treatment technique**: applying nanocatalysts with reusability study can enable pollutant degradation, increase the hydroxyl radical production, and promote the process selectivity.
- **Environmental impact assessment**: analyzing the potential byproducts hazardous, long-term ecosystems impacts and monitoring the sustainability of treatment technology.
- **Process modeling and simulation**: applying artificial intelligence will enable the accurate prediction of the whole pollutant degradation process.
- **Circular economy and resource recovery**: recovery of valuable compounds from industrial wastewater promote more sustainable and economically viable treatment approaches.

## Data Availability

All data generated or analyzed during this study are included in this published article.
